# Characterization of the Core and Caste-Specific Microbiota in the Termite, *Reticulitermes flavipes*

**DOI:** 10.3389/fmicb.2016.00171

**Published:** 2016-02-17

**Authors:** Jacquelynn Benjamino, Joerg Graf

**Affiliations:** ^1^Department of Molecular and Cell Biology, University of Connecticut, StorrsCT, USA; ^2^Institute for Systems Genomics, University of Connecticut, StorrsCT, USA

**Keywords:** *Reticulitermes flavipes*, Core microbiota, Termite caste microbiota, 16S rRNA gene, Illumina amplicon sequencing

## Abstract

The hindgut of the termite *Reticulitermes flavipes* harbors a complex symbiotic community consisting of protists, bacteria, and archaea. These symbionts aid in the digestion of lignocellulose from the termite’s wood meal. Termite hindguts were sampled and the V4 hyper-variable region of the 16S rRNA gene was sequenced and analyzed from individual termites. The core microbiota of worker termites consisted of 69 OTUs at the 97% identity level, grouped into 16 taxa, and together accounted for 67.05% of the sequences from the bacterial community. The core was dominated by *Treponema*, which contained 36 different OTUs and accounted for ∼32% of the sequences, which suggests *Treponema* sp. have an important impact on the overall physiology in the hindgut. Bray–Curtis beta diversity metrics showed that hindgut samples from termites of the same colony were more similar to each other than to samples from other colonies despite possessing a core that accounted for the majority of the sequences. The specific tasks and dietary differences of the termite castes could have an effect on the composition of the microbial community. The hindgut microbiota of termites from the alate castes differed from the worker caste with significantly lower abundances of *Treponema* and *Endomicrobia*, which dominated the hindgut microbiota in workers and soldiers. Protist abundances were also quantified in the same samples using qPCR of the 18S rRNA gene. *Parabasalia* abundances dropped significantly in the winged alates and the *Oxymonadida* abundances dropped in both alate castes. These data suggest that the changes in diet or overall host physiology affected the protist and bacterial populations in the hindgut. The in-depth bacterial characterization and protist quantification in this study sheds light on the potential community dynamics within the *R. flavipes* hindgut and identified a large and complex core microbiota in termites obtained from multiple colonies and castes.

## Introduction

Termites have long been studied because of their uncommon diet and complex hindgut microbiota. Researchers study the termite symbiotic system for the discovery of lignocellulases to aid in biofuel production (Tartar et al., 2009; Sethi et al., 2013), understanding of the coevolution of the host and symbionts (Hongoh et al., 2005), and the ability to manipulate and study the structure and function of a complex microbiota (Brauman et al., 2001). Termites are descendants of the wood-feeding cockroach *Cryptocercus*, and are separated into two groups, higher and lower termites (Dietrich et al., 2014). Lower termites contain an abundance and diversity of flagellate protozoa that aid them in the digestion of wood and higher termites have been reported not to harbor symbiotic protists (Ohkuma, 2003); however, recently, a low-abundant ciliate has been detected in the guts of several higher termites species (Rahman et al., 2015).

*Reticulitermes flavipes*, the Eastern subterranean termite, is indigenous to the northeastern United States and harbors a tripartite symbiosis in its hindgut consisting of protozoal, bacterial, and archaeal symbionts (Ohkuma, 2003). The digestive enzymes from *R. flavipes* cannot fully break down the lignocellulosic components of wood, the termites’ sole food source, while the hindgut symbionts aid the digestion of these wood particles and provide acetate as a nutrient for the host (Ohkuma, 2003). The composition of the microbiota residing in the hindgut of *R. flavipes* has been previously investigated using culture-independent approaches as reviewed by Scharf (Scharf, 2015). Other studies investigated the *R. flavipes* hindgut microbiota by pooling DNA from several termites and sequencing a variable region of the 16S rRNA gene using 454 pyrosequencing (Ohkuma, 2008). Boucias et al. (2013) reported that the community is comprised of an estimated 581 bacterial operational taxonomic units, OTUs at the 97% identity level with approximately 80% of the symbionts belonging to the phyla *Spirochaetes*, *Elusimicrobia*, *Bacteroidetes*, *Firmicutes*, and *Proteobacteria*. They also evaluated the maintenance and stability of the microbial community in the hindgut and discovered that after *R. flavipes* termites were fed either a lignocellulose or cellulose diet for seven days, 88% of the OTUs in the hindgut microbiota were preserved despite the different diets, while only 12% of the OTUs were variable (Boucias et al., 2013). Proctodeal feeding has been suggested as an important mechanism contributing to this stability of the microbial community wherein the worker caste feeds the other members in the colony via fecal transfer (Buczkowski et al., 2007).

The core microbiota is defined as the organisms shared across multiple samples obtained from the same host, which is likely to play crucial roles in the functionality of that habitat (Turnbaugh et al., 2007). The core community of any symbiotic system is important in the health and maintenance of the symbiosis. Many studies have found the presence of a core microbiota in a variety of hosts, either in the form of a taxonomic core or a functional core (encoded genes) (Huse et al., 2012; Shade and Handelsman, 2012; Turnbaugh and Gordon, 2013; Maltz et al., 2014). Knowing the composition of the core microbiota is important because it ensures the maintenance of functions within the habitat and serves as an anchor for community resistance and/or resilience (Huse et al., 2012; Shade et al., 2012). However, it should be noted that differences in the hindgut microbiota can be critical for nestmate recognition or various caste-related functions (Cleveland, 1925; Minkley et al., 2006). Determining the core in smaller animals such as insects can be more challenging as the samples can be very small and thus some tend to pool samples prior to DNA extraction. While these studies provide important insight into the complexity and stability of the community, pooling samples averages the signal and prevents detection of variation between individuals. As the resulting OTU data is averaged, determining the prevalence in the individuals comprising the sample is impossible, and thus the core microbiota cannot be accurately determined (Hamady and Knight, 2009). These studies still provide valuable information but are distinct from a “core” and we will refer to such conclusions as the common microbiota in this manuscript. The core microbiota in some insects is extremely small, for example the core consists of ten OTUs in the bed bug *Cimex lectularius* (Meriweather et al., 2013), two taxa in *Anopheles gambiae* (Wang et al., 2011), and nine taxa in the honey bee (Moran et al., 2012; Sabree et al., 2012). The common microbiota of the fungus-growing higher termite, *Macrotermitinae*, (Otani et al., 2014) non fungus-growing higher termites, lower termites and cockroaches is shared between eleven phyla residing in all four groups and the five most abundant phyla being *Firmicutes*, *Bacteroidetes*, *Spirochaetes*, *Proteobacteria*, and *Synergistes* (Dietrich et al., 2014; Otani et al., 2014).

We hypothesize that the feeding habits dictate protist abundance, which in turn affects the abundance of protist-associated bacteria such as *Endomicrobia* and *Treponema*. Caste specific micrbiotas have been shown in the honey bee where queens have a higher abundance of *Parasaccharibacter apium* (Kapheim et al., 2015), *Alphaproteobacteria* and a *Firmicute* (Firm-5) (Tarpy et al., 2015), while workers harbor a higher abundance of *Betaproteobacteria* and *Gammaproteobacteria* (Tarpy et al., 2015). Being a eusocial insect, *R. flavipes* colonies have a caste system made up of juveniles, workers, soldiers, and reproductives. The workers forage for food and return to the nest to feed other members. The soldiers’ sole purpose is to defend the colony and these individuals have an enlarged mandible, which makes it impossible for them to masticate wood (Cleveland, 1925). Select members of the worker caste morph into alates (winged termites) and harbor a dramatically reduced number of protists in their gut while preparing to swarm (Shimada et al., 2013). After which they lose their wings, pair up and become reproductive termites that establish new colonies. The microbiota in the alates is of particular importance as these termites are the reproductive caste that found new colonies and presumably are the source of the key members of the hindgut microorganisms unless they can be acquired from the environment. The king and queen reproduce for colony growth, and are also fed by the worker caste. The soldier and reproductive castes are thought to have a reduced need for hindgut protists as they do not partake in the initial breakdown of wood into its constituent parts. Therefore, these castes have fewer protists in the hindgut, while kings and queens in mature colonies have no hindgut protists (Shimada et al., 2013).

While researchers are trying to understand the functions of the termite symbionts, fundamental aspects about the microbiota are not well known including the variability in the composition of microbiota between colonies, between different castes and between individual workers obtained from the same colony. We characterized the microbiota by sequencing the V4 region of the 16S rRNA gene from the hindguts of individual *R. flavipes* obtained from multiple colonies. Our analysis revealed a stable microbial community within the hindgut of workers that is comprised by a large core community, while there were significant differences in the abundance of protists and in the composition of the bacterial community in different castes.

## Materials and Methods

### Termite Collection and Maintenance

*Reticulitermes flavipes* termites were either collected using cardboard traps about a month after placement, captured directly from a rotting log, or purchased. The locations of termite colonies at time of collection are as follows: Mansfield CT (CT.A, October 2011 & CT.C, August 2012), Willington CT (CT.B, October 2013), Willimantic CT (CT.D, July 2013), Groton MA (MA.B, July 2013), Woods Hole MA (MA.C, July 2013), or purchased from Connecticut Valley Biological Supply Co. in Southampton MA (MA.A, June 2013). Additional alate termites used in the qPCR assays for the caste analyses were collected from an eighth colony (April 2014, Storrs, CT, USA), along with workers from the same colony for comparison. Once in the lab, the termites were placed in plastic containers with moist, autoclaved sand and spruce. Colonies were maintained at room temperature in the dark, and the sand was moistened with water every 3–4 weeks. Each colony, with the exceptions of CT.A and CT.C, were sampled upon collection. Colonies CT.A and CT.C were sampled for 4 months following the collection date. Termites sampled were assumed to have been initially collected from the natural habitat, as no evidence of reproduction was observed during the maintenance of the colonies in the lab.

### Molecular Identification of Termites

Termite DNA from each colony was used for sequencing the Cytochrome Oxidase II (COII) gene to ensure the termites were *R. flavipes* (**Supplementary Figure 1**). Primers used for COII sequencing were a modified version of A-tLEU (5′-CAGATAAGTGCATTGGATTT-3′) and B-tLYS (5′-GTTTAAGAGACCAGTACTTG-3′) from Liu and Beckenbach (1992). Sequences were aligned in Geneious 6.1.7 using a MUSCLE alignment (Kearse et al., 2012). A neighbor-joining consensus tree was created with 100 bootstraps iterations, using COII sequences from this study along with sequences from multiple *Reticulitermes* species from NCBI (accession numbers: KR537205-12, JF7962324.1, KM245774-5, JF796221-2, AF262607.1, AY808093.1, EU253889.1, FJ806884.1, JQ280728-36, JX142171-72, JX142149-54) (Su et al., 2006; Legendre et al., 2008; Lim and Forschler, 2012; Perdereau et al., 2013).

### Sample Collection and DNA Isolation

Hindguts were removed from the termite by pulling the thorax and anus apart with forceps (Matson et al., 2007) and placed in TE buffer (10 mM Tris-HCl, 1 mM EDTA, pH 8.0). Samples consisted of single, whole hindguts with the exception of the data for colony CT.A where each data point represents pools from five hindguts (these samples were collected before we had established an efficient methods for single hindgut sampling). Seven colonies were sampled for all analyses, excluding the caste analysis. The number of samples per colony are as follows: CT.A (11 samples), CT.B (5), CT.C (8), CT.D (4), MA.A (9), MA.B (6), MA.C (2). For the caste analysis, 19 samples were taken from seven colonies; soldiers and alates were always matched with workers from the same colony. The number of samples per caste are as follows: workers (nine samples), soldiers (5), winged alates (2), de-winged alates (3). DNA was isolated immediately after collection using a modified (the starting lysis buffer was 500 μL and the final elution volume was 30 μL AE buffer) RBB+C isolation protocol as described by Yu and Morrison (2004). This method uses repeated bead beating along with chemical and high temperature cell lysis, and DNA precipitation followed by RNA and protein removal using a QIAmp DNA Mini Kit (Qiagen^®^, Germantown, MD, USA) column.

### PCR Amplification of 16S rRNA Gene and Library Preparation

Hindgut samples were amplified using the V4 hyper-variable region of the 16S rRNA gene using primers developed by Caporaso et al. (2012). PCR reactions included Phusion^®^ High-Fidelity PCR Master Mix with HF Buffer (New England Biolabs Inc., Ipswich, MA, USA) (50% of total volume), 10 μM forward and reverse primers, ∼10 ng DNA, and dH_2_O to the final volume of 25 μL. All reactions were amplified in triplicate using the following parameters: 94°C for 3 min, followed by 30 cycles of 94°C (45 s), 50°C (60 s), and 72°C (90 s), with a final extension of 72°C for 10 min (Nelson et al., 2014). Triplicate reactions were pooled and each sample was tested for size by running a 1% agarose gel.

Amplicons were purified and size selected using Agencourt AMPure XP (Beckman Coulter Inc., Brea, CA, USA) magnetic beads (0.65 × μL of sample volume) to select for 400 bp amplicons according to manufacturer’s protocol. Samples were then quantified using a Qubit^®^ dsDNA HS Assay (ThermoFisher Scientific Inc.). Concentrations of each sample were calculated and then diluted to 4 nM. All samples were pooled in equimolar amounts for sequencing.

### Sequencing and Data Processing

Samples were sequenced using an Illumina MiSeq (Illumina, San Diego, CA, USA) with custom sequencing primers added to the reagent cartridge (Caporaso et al., 2012) and sequenced 2 × 150 bp (CT.A & CT.C) or 2 × 250 bp. Both sequencing methods sequenced the entire V4 region of the 16S rRNA gene and the same merging and quality control parameters were used on both sets of data. The reads were processed as described previously by Nelson et al. (2014). Briefly, output reads were merged to create single reads spanning the entire 254 bp of the V4 hypervariable region using SeqPrep^1^, and the PhiX control reads were removed by mapping to the PhiX genome. Data analysis was performed using QIIME (Caporaso et al., 2010). Low quality reads (less than Q30) were removed and operational taxonomic units (OTUs) were determined by clustering reads to the Greengenes reference 16S rRNA gene reference dataset (2013-08 release) (DeSantis et al., 2006) at a 97% identity, and then performing *de novo* OTU clustering on reads that failed to cluster to a reference (McDonald et al., 2011; Nelson et al., 2014). Chimeras were then removed and the dataset was filtered to remove singleton and doubleton OTUs and then OTUs present at less than 0.0005% (Bokulich et al., 2012). The data was rarified to 15,000 reads per sample in order to include all samples in this study.

### Data Analysis

The core microbiota was determined using all of the samples collected from individual *R. flavipes* workers in this study (excluding colony CT.A). Using QIIME, with Greengenes (2013-08) and the DictDb database (Mikaelyan et al., 2015), we calculated the OTUs (at the 97% identity level) that were present in at least 95% of the samples (Huse et al., 2012). These OTUs were then paired with taxonomy to the lowest level of classification, and the sequence abundance of each core OTU was reported. The sequences in the DictDb database were shortened to only include the V4 hypervariable region and combined with the Greengenes database. The combined file was aligned to generate the aligned reference. Some sequences failed to align due to shortness in length and were removed from the unaligned reference file (5,845 sequences out of 55,394).

The samples used in the geographic analysis were all from the worker caste. After quality filtering and rarifying to 15,000 sequences per sample, alpha diversity (Shannon and Phylogenetic diversity) (Faith and Baker, 2006) and beta diversity metrics (Bray–Curtis) (Lozupone and Knight, 2005; Lozupone et al., 2006; Anderson et al., 2011) were performed using QIIME 1.8 and R 3.2.0 (R Development Core Team, 2005; Wickham, 2009; Oksanen et al., 2015). The PERMANOVA statistical analysis was performed to determine the significance of microbial community differences among the different colonies using the Bray–Curtis dissimilarity matrix in QIIME (Caporaso et al., 2010). This analysis was performed over 999 permutations and returned a Pseudo-F (f) statistic along with a *p*-value *(p)*.

*Reticulitermes flavipes* worker, soldier, and alate hindgut samples collected from various colonies were used in the analysis of the caste microbiota. For each non-worker (soldier, alate), a worker was collected at the same time from the same colony for comparison. The winged alates were collected on February 27, 2013, and de-winged alates were collected on May 31, 2013. The bacterial taxonomic abundances were averaged for each caste, and the averages were used in the analysis. Similar to the statistical analysis done on the microbial communities from different colonies, the PERMANOVA statistical analysis was used to determine the significance of the microbial community differences between workers and soldiers, workers and winged alates, and workers and de-winged alates. The sequences (OTU assignments using Greengenes) in the caste dataset were compared to the DictDb database, which is a curated database for microbes from termites and cockroaches that provides greater taxonomic resolution, using BLASTN at the 97% identity level (Altschul et al., 1990; DeSantis et al., 2006; Mikaelyan et al., 2015). *Spirochaete* sequences with 100% query coverage were assigned OTUs and taxonomy using the DictDb database. *Treponema* sequences that did not match reference sequences in the DictDb database or Greengenes database were designated as ‘*de novo’*. A one-way ANOVA with a Bonferroni post-test was performed for each taxonomic grouping of *Spirochaetes* using GraphPad Prism version 6.0f for Mac OSX^2^ (GraphPad Software, San Diego, CA, USA).

### Quantitative PCR (qPCR) of Protist Symbionts

Caste hindgut samples with 16S rRNA sequencing data were used for qPCR analysis. Additional alate samples (and workers from the same group) were added to the analysis, the number of samples per caste are: workers (19 samples), soldiers (3), winged alates (12), de-winged alates (6). Primer sets for the two groups of protists found in the hindgut were designed using 18S rRNA gene sequences from NCBI in Geneious (phylum *Parabasalia* and order *Oxymonadida*) (Kearse et al., 2012). The primers were then tested on hindgut contents, termite DNA, and bacterial DNA to ensure there was no amplification of termite or bacterial DNA. Primer sequences are as follows: Para361F-5′CGCGAAACTTACCCACTCG-3′, Para510R-5′TTACCGCAGCTGCTGGC-3′ and Oxy161bF-5′CGGATAGCCGTAGTAATTCTAGAGCT-3′, Oxy352bR-5′AACGTCAGGTTGATAGGTTAGAAATT-3′. All reactions were setup in a 10 μL volume including: SsoAdvanced SYBR Green Supermix (Bio-Rad Laboratories Inc., Hecrules, CA, USA) (50% of reaction volume), 10 μM forward and reverse primers (15% each of reaction volume), dH_2_O (10% of reaction volume) and 1 μL of DNA template. Reactions were amplified in triplicate using a CFX96 Real-Time Thermocycler (Bio-Rad Laboratories Inc., Hercules, CA, USA) with the following parameters for the *Oxymonadida*: 95°C (3 min), followed by 40 cycles of 95°C (30 s), 64°C (30 s), and 72°C (30 s). The parameters for the *Parabasalia* were the same except that the annealing temperature was 67°C. Negative controls with no template added were prepared and tested with each set of reactions. Standard curves were generated for each primer set using 10^2^–10^8^ copies per reaction and the real-time data was normalized to the concentration of DNA added to the PCR reaction to calculate the *C*_t_ value, representing a single hindgut and then square-root transformed for statistical analyses. A one-way ANOVA with a Bonferroni post-test was performed for each caste in both protist groups using GraphPad Prism version 6.0f for Mac OSX (GraphPad Software, San Diego, CA, USA).

### Data Availability

The 16S rRNA gene sequence data was deposited in the European Nucleotide Archive (ENA) SRA under project ID PRJEB5527.

The COII gene sequence data was deposited in GenBank under accession numbers: KR537205-12.

## Results

### Identifying the Core Microbiota of *R. flavipes*

**FIGURE 1:**
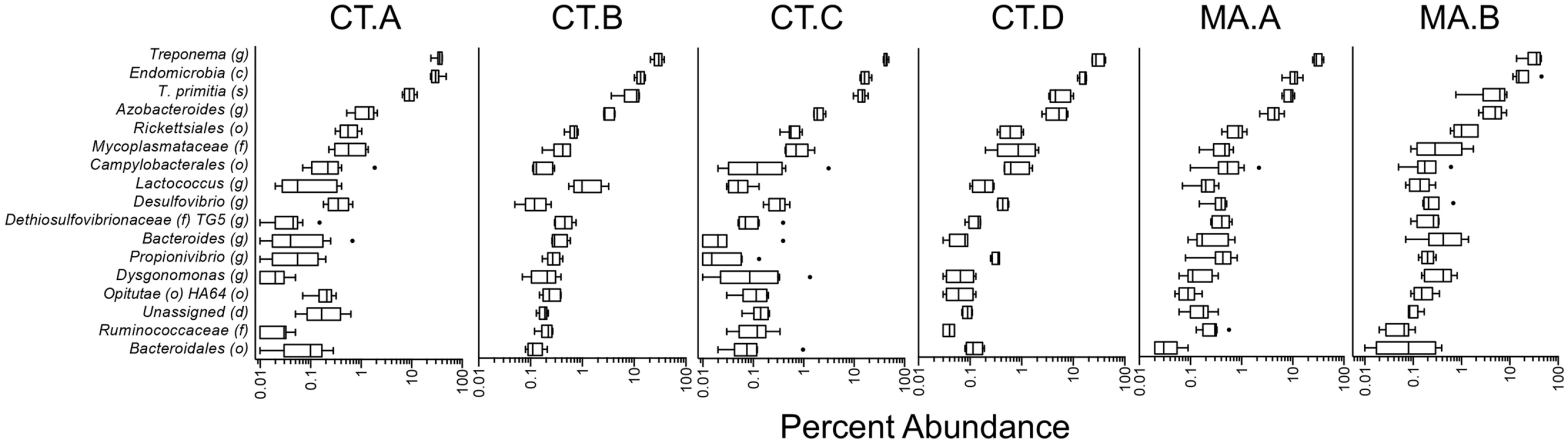
**The abundance of core taxa in the worker hindgut.** A box and whisker plot for each colony is shown with the abundance of the taxa found in the core microbiota. The abundance of each taxon is consistent throughout the six. The number of samples in each colony are as follows: CT.A (*n* = 11), CT.B (*n* = 5), CT.C (*n* = 8), CT.D (*n* = 4), MA.A (*n* = 9), MA.B (*n* = 6). Colony MA.C was not used in this analysis due to its small sample size (*n* = 2). Colony CT.A contained five pooled hindguts per sample and was not used in determining the core microbiota, however, it showed the same pattern of core taxon abundances.

Determining a core microbiota is important for any host-associated or environmental community because one can infer the composition of the “healthy” or undisturbed community and a diseased one (Turnbaugh et al., 2007; Shade and Handelsman, 2012). In our study, we defined the core microbiota as the OTUs at the 97% identity level that were present in 95% of the samples (Huse et al., 2012). Our data showed that the core of the worker termites consisted of 69 OTUs and accounted for 67.05% of the sequences of the hindgut microbiota (**Table 1**). Of these OTUs, the genus *Treponema*, contained 41 OTUs, 5 of them being classified to the species *T. primita*, and accounted for almost 41.43% of the total sequences in the hindgut. The class *Endomicrobia* (8 OTUs) and genus *Azobacteroides* (3 OTUs) had an abundance of 18.50 and 3.18%, respectively. The remaining 17 OTUs fell into 13 taxa and comprised 3.95% of sequences from the hindgut. 32.95% of OTUs found in the termite hindgut varied between individuals and were not considered to be part of the core microbiota. One OTU (0.16%) was identified from the DictDb (cockroach and termite specific) database and was unassigned. Box and whisker plots showing taxon abundances (**Figure 1**) were created for each colony, using the taxa found in the core microbiota. These data support the average taxon abundances shown in the core (**Table 1**). All colonies show similar abundance patterns for each of the core taxa.

**Table 1:** The *R. flavipes* worker core hindgut microbiota.^a^

Core taxonomy			
		
Class	Taxon^b^	Number of OTUs in taxon	Average abundance
*Spirochaetes*	*Treponema (g)*	36	32.64%
*Endomicrobia*	*Endomicrobia(c)*	8	18.50%
*Spirochaetes*	*primitia (s)*	5	8.79%
*Bacteroidia*	*Azobacteroides (g)*	3	3.18%
*Alphaproteobacteria*	*Rickettsiales (o)*	4	0.74%
*Mollicutes*	*Mycoplasmataceae (f)*	1	0.61%
*Epsilonproteobacteria*	*Campylobacterales (o)*	1	0.44%
*Bacilli*	*Lactococcus (g)*	1	0.39%
*Deltaproteobacteria*	*Desulfovibrio (g)*	1	0.32%
*Synergistia*	*TG5 (g)*	1	0.25%
*Bacteroidia*	*Bacteroides (g)*	1	0.24%
*Betaproteobacteria*	*Propionivibrio (g)*	1	0.22%
*Bacteroidia*	*Dysgonomonas (g)*	1	0.19%
*Opitutae*	*HA64 (o)*	2	0.16%
*Unassigned*	*Unassigned (d)*	1	0.16%
*Clostridia*	*Ruminococcaceae (f)*	1	0.12%
*Bacteroidia*	*Bacteroidales (o)*	1	0.11%

	**Total**	**69**	**67.05%**


***^a^**The *R. flavipes* core microbiota includes taxa in which the OTUs (at the 97% identity level) were found in at least 95% of the samples sequenced.*

***^b^**Taxonomic level is designated in parentheses as: (d) domain, (p) phylum, (c) class, (o) order, (f) family, and (g) genus. Taxonomic level listed for each organism is the lowest classification available.*

### Analysis of the Hindgut Microbiota Among Different Colonies

**FIGURE 2:**
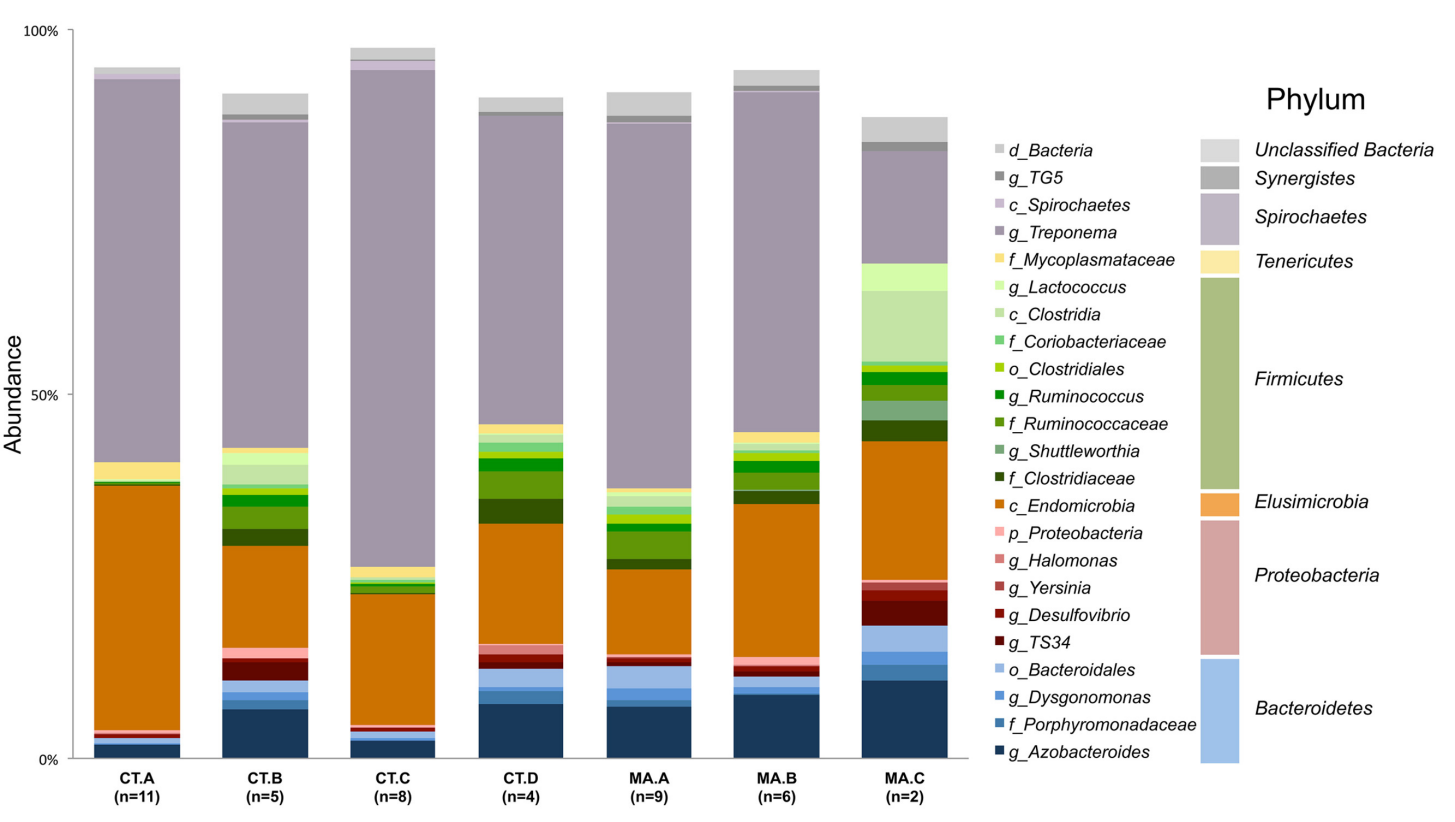
**Composition of the microbiota in the *R. flavipes* worker hindgut.** The genus *Treponema* and class *Endomicrobia* dominate the hindguts in all colonies. Taxonomic abundances from hindgut samples of the same colony were averaged together. Taxa present at a sequence abundance of 1% or higher are shown. Letters before the taxonomic name represent the taxonomic level at which that organism was identified (d-domain, c-class, f-family, o-order, g-genus).

The microbiota of xylophagus insects, such as the wood-feeding cockroach, *Cryptocercus kyebangensis*, is shared between members of the colony through proctodeal trophalaxis (Park et al., 2002). This process is thought to create a homogenous microbial community throughout the colony, which may aid in digestion and colony health. To determine the homogeneity of the *R. flavipes* hindgut microbiota, individual and pooled worker hindguts were sampled from seven colonies originating in Massachusetts or Connecticut. Alpha diversity analyses were performed and the values for each sample within a colony averaged and a single value was presented for each grouping (**Table 2**). The Shannon index and equitability show that the microbial community is not evenly distributed. In the case of colonies CT.A and CT.C (sampled over 4 months in the lab), the microbial community becomes less complex over time (One-way ANOVA; (*F*_(6,38)_ = 36.54, *p* < 0.0001)). The phylogenetic diversity differs among the microbial communities in different colonies, with some having a less diverse microbiota than others. At the phylum level, *Spirochaetes* dominate the hindgut community, with an average sequence abundance of 55% among the 45 *R. flavipes* workers. *Elusimicrobia* and *Bacteroidetes* are present at 24 and 10%, respectively. The remaining 11% of sequences belong to the phyla *Proteobacteria*, *Firmicutes*, *Tenericutes*, and *Synergistes*, or were unclassified *Bacteria* (**Figure 2**, **Supplementary Figure 2** and **Supplementary Table 1**). In our study, the archaeal community was present at an abundance of less than 0.1%. This could be due to the specificity of the primers used for amplifying the V4 region of the 16S rRNA gene, but other studies using different approaches also reported that in lower termites, archaea are present at low abundances (Berchtold et al., 1999; Hongoh, 2010).

**Table 2:** Bacterial alpha diversity of the *R. flavipes* worker hindgut among different colonies based on the 16S rRNA amplicon.

Colony^∗^	*n*^+^	Shannon index (H′)	Shannon equitability (E_H_)	Phylogenetic diversity (PD)
CT.A	11	3.54 ± 0.16	0.62 ± 0.02	48.25 ± 3.11
CT.B	5	4.57 ± 0.17	0.72 ± 0.02	88.26 ± 2.40
CT.C	8	3.62 ± 0.27	0.62 ± 0.03	55.16 ± 9.53
CT.D	4	4.56 ± 0.19	0.74 ± 0.03	72.40 ± 3.40
MA.A	9	4.70 ± 0.23	0.73 ± 0.03	92.22 ± 2.70
MA.B	6	4.39 ± 0.31	0.71 ± 0.04	70.40 ± 5.52
MA.C	2	4.48 ± 0.21	0.73 ± 0.02	79.30 ± 6.79


*^∗^Colony name denotes which state the colony derived from, Connecticut (CT) or Massachusetts (MA).*

*^+^n represents the number of termites sampled from each colony.*

**FIGURE 3:**
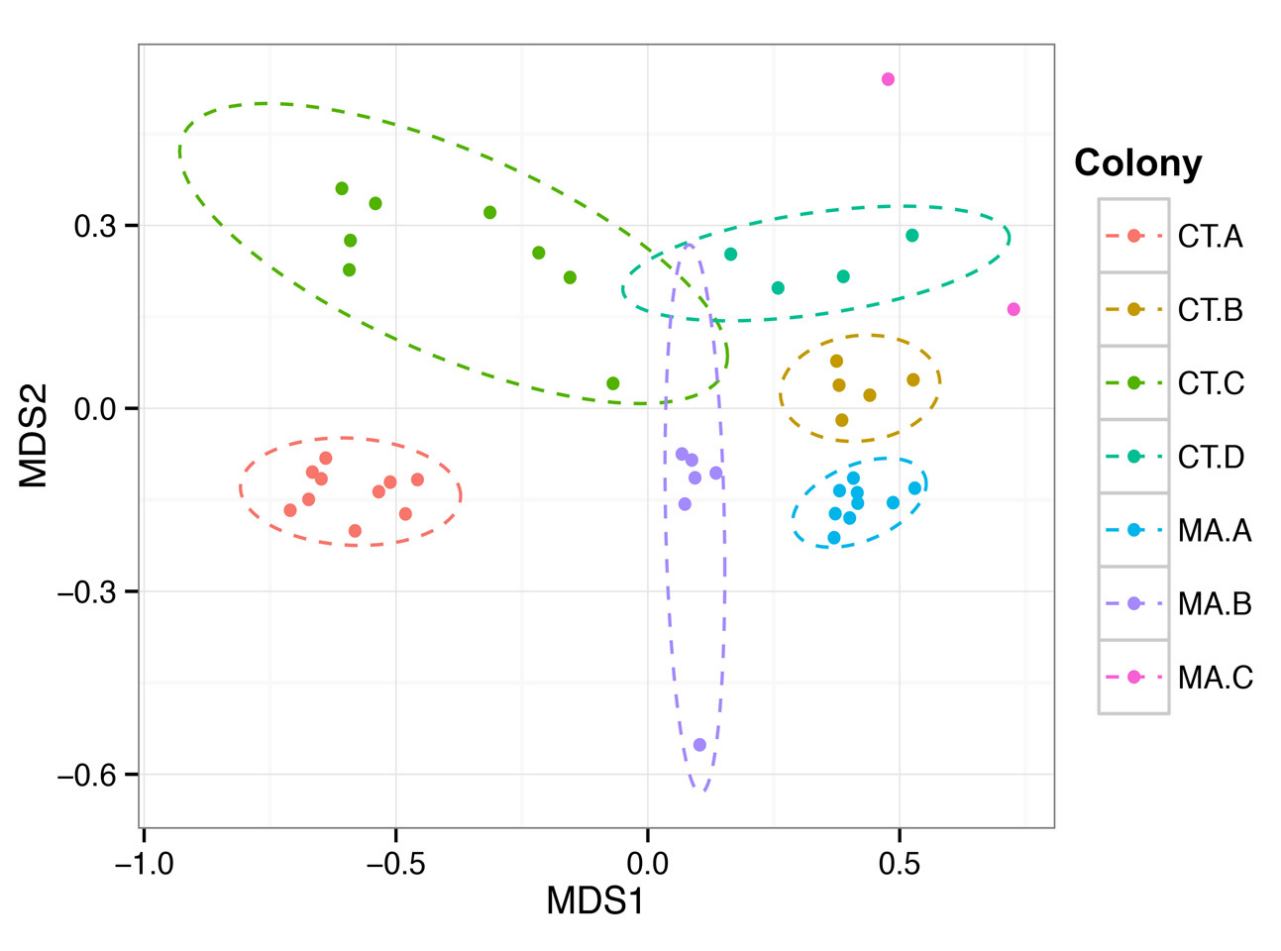
**Colony specificity of the *R. flavipes* worker hindgut microbiota.** The hindgut microbiota shows more similarity between hindguts from the same colony than between colonies (PERMANOVA, *F* = 6.6201, *p* = 0.001). Nonmetric Multi-Dimensional Scaling was performed on the hindgut microbiota from different colonies using the Bray–Curtis dissimilarity metric. The ellipses were obtained from the standard deviation within a colony, and plotted using a 95% confidence level. The differences in the MA.C colony could be due to the coastal environment compared to the inland environment of the other colonies.

We wanted to assess whether the microbial community residing in the hindgut of one termite was more similar to other termites in the same colony than to termites from a different colony in similar geographic locations. The Bray–Curtis beta diversity analysis was performed to determine similarities and differences in the composition of the microbiota and was used to create a NMDS (non-metric multi-dimensional scaling) plot of the 45 hindgut samples (**Figure 3**). The microbial communities within a colony grouped significantly closer together than to communities from other colonies (PERMANOVA, *f* = 8.62, *p* = 0.001) and there was no clustering of samples according to the state from which they originated nor according to the COII sequence of the termite.

### Comparison of the Microbiota Among Different Castes

A termite colony is composed of various castes, each with a unique function contributing that might influence the composition of the hindgut microbiota (Lewis and Forschler, 2013). Alpha diversity analysis of the members from each caste was performed, and the evenness and richness of the microbial community in each caste were similar (**Table 3**). While a trend of a difference in the phylogenetic diversity of each caste was noted, it was not statistically significant, perhaps future studies with a larger sample size might support this finding.

**Table 3:** Bacterial alpha diversity of the *R. flavipes* hindgut among different castes based on the 16S rRNA amplicon.

Caste	*n*^+^	Shannon index (H′)	Shannon equitability (E_H_)	Phylogenetic diversity (PD)
Workers	9	4.14 ± 0.48	0.68 ± 0.06	68.44 ± 9.11
Soldiers	5	3.86 ± 0.25	0.65 ± 0.03	56.70 ± 6.88
Winged alates	2	3.17 ± 1.49	0.56 ± 0.19	46.46 ± 25.18
De-winged Alates	3	3.73 ± 0.12	0.63 ± 0.02	61.89 ± 3.25


*^+^n represents the number of termites sampled from each caste.*

**FIGURE 4:**
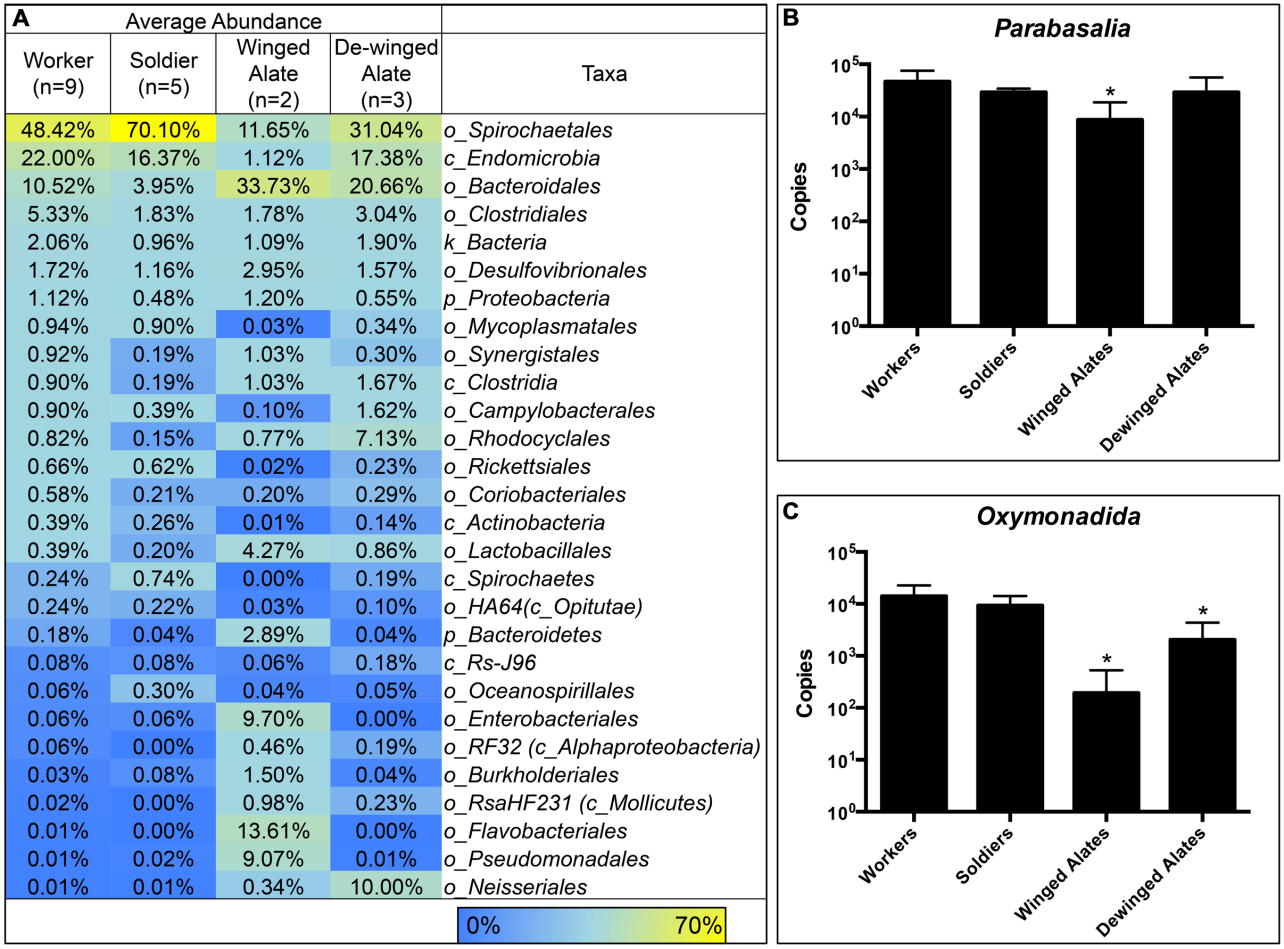
**Comparison of Bacterial Taxa and Protists in *R. flavipes* Castes.** *Treponema*, *Endomicrobia*, and protist abundances are lower in the alate castes compared to the worker caste. Hindgut samples were taken from worker and non-worker termites in various colonies. **(A)** Abundances for each caste were averaged and compared to the worker caste. In winged alates, the abundance of the two dominating taxa, *Spirochaetales*, and *Endomicrobia*, are lower in abundance compared to the worker caste. *Enterobacteriales*, *Flavobacteriales*, *Pseudomonadales*, and *Neisseriales* are present in very low abundances in the workers and soldiers, but were more abundant in the alate caste. Protists belonging to the phylum *Parabasalia* **(B)** and the order *Oxymonadida* **(C)** were quantified using qPCR. Each caste was compared to the worker caste using a one-way ANOVA. ^∗^ Indicates a significant value compared to the worker caste. **(B)** *Parabasalia* protists were less abundant in the winged alates [*F*_(3,36)_ = 11.9, *p* < 0.0001]. **(C)** *Oxymonadida* protists were less abundant in the winged and de-winged alates compared to workers [*F*_(3,36)_ = 36.94, *p* < 0.0001].

**FIGURE 5:**
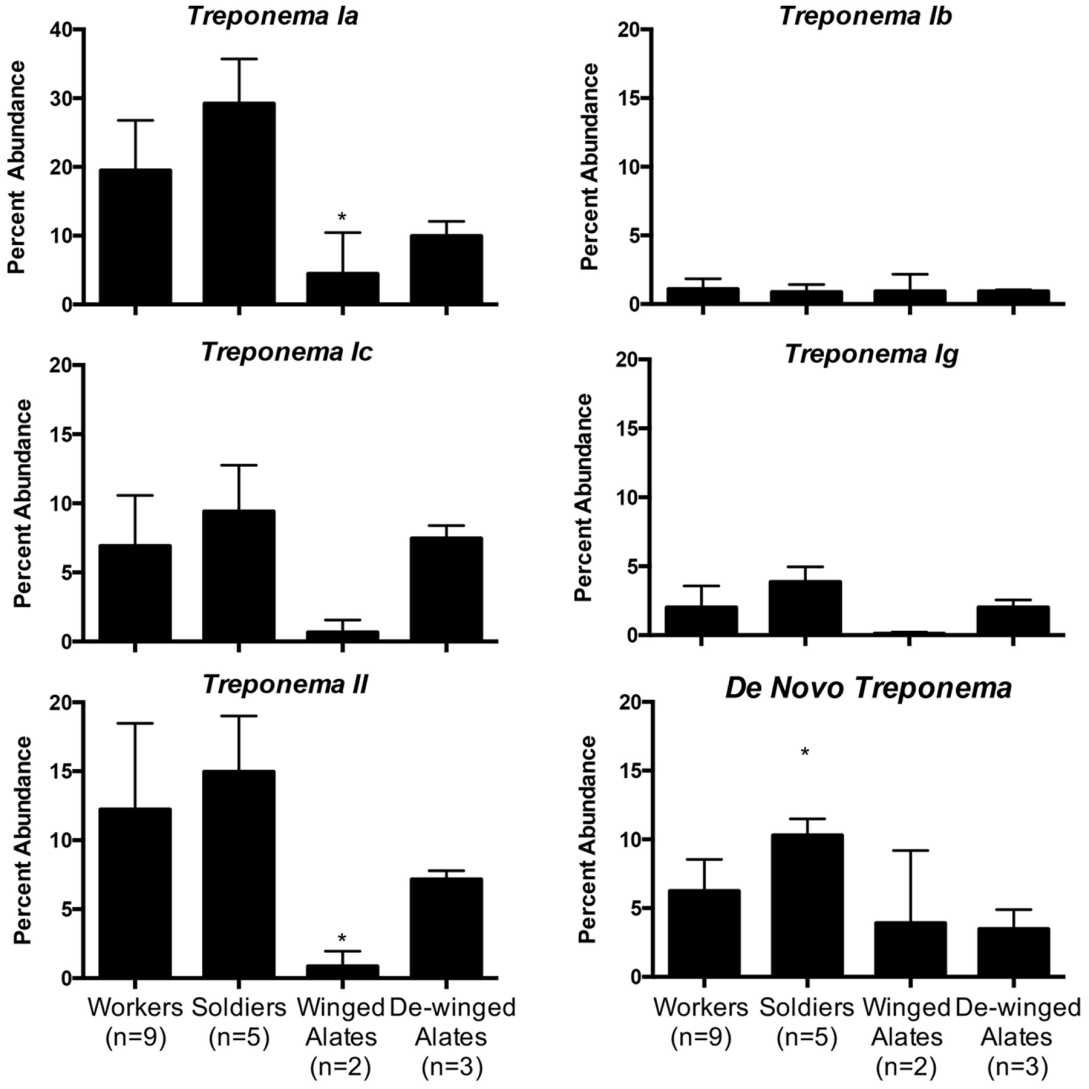
**Relative abundance of *Treponema* groups among different castes.** Five groups of *Treponema* differ in abundance in workers, soldiers, winged alates, and de-winged alates. OTUs classified as *Treponema* were categorized into groups Ia, Ib, Ic, Ig, and II according to the DictDb database. OTUs that could not be classified in DictDb were processed *de novo* and classified to the genus *Treponema*. *Treponema Ia* is the most abundant group while *Treponema Ib* is the least abundant but most consistent throughout the castes. *Treponema Ia* and *II* have significantly lower abundances in winged alates compared to workers as indicated by a one-way ANOVA [*F*_(3,15)_ = 9.331, *p* = 0.001, and *F*_(3,15)_ = 4.489, *p* = 0.019]. *De Novo Treponema* have a significantly higher abundance in soldiers compared to workers as indicated by a one-way ANOVA [F(3,15) = 7.043, p = 0.0035]. All significant data are indicated by an asterisk ^∗^.

Each caste is known to have different diets and perform specialized functions in the colony, which suggest that the hindgut microbiota may reflect these differences (Lewis and Forschler, 2013). Averaging the sequence abundances at the taxonomic order level for each caste and comparing the values to the worker caste enabled a comparison of the microbial composition in different castes. We found differences in taxonomic abundances between alates and workers according to a PERMANOVA (winged: *f* = 3.59, *p* = 0.001; de-winged: *f* = 2.27, *p* = 0.01). Sequences representing the two dominating taxa, order *Spirochaetales* and class *Endomicrobia*, decreased in abundance in the winged alates from 48 and 22% to 11.6 and 1.1%, respectively, (**Figure 4** and **Supplementary Figure 3**). Sequences belonging to the order *Bacteroidales* were found in the workers and soldiers at an abundance of less than 11% while they were present at over 20% in both the winged and de-winged alates. The orders *Enterobacteriales*, *Flavobacteriales*, and *Pseudomonadales* were present at average abundances of 9.7, 13.6, and 9%, respectively, in the winged alates while they were below the limit of detection in workers, soldiers, and de-winged alates (0–0.06%). The *Spirochaete* sequences were further classified using OTU assignments and taxonomic classifications from the DictDb database (**Figure 5**) (Mikaelyan et al., 2015). The sequences were classified into five groups: *Treponema* Ia, Ib, Ic, Ig, II, and sequences that were not similar to any in the DictDb database were labeled as *de novo Treponema*. The subgroup *Treponema* Ia were the most abundant taxon in the hindgut with relative abundances reaching up to 33%. *Treponema* Ib were the least abundant and most consistent subgroup with average abundances of 1% for all castes. The abundances of *Treponema* Ia [*F*_(3,15)_ = 9.331, *p* = 0.001] and *Treponema* II [*F*_(3,15)_ = 4.489, *p* = 0.0190] were significantly lower in the winged alates when compared to the worker caste (one-way ANOVA with Bonferonni-corrected *p*-values), which may indicate that *Treponema* Ia and II are necessary in the digestion process of workers and associated with protists.

*Endomicrobia*, along with some *Spirochaetes* are known protist symbionts in the termite hindgut (Iida et al., 2000; Stingl et al., 2005; Ikeda-Ohtsubo et al., 2007). The observed decrease in the abundance of these bacteria in winged alates led us to investigate the protist abundances in the same samples. Abundances of two groups of protists, phylum *Parabasalia* and order *Oxymonadida*, were determined using qPCR. The protist abundances in each caste were compared to the worker caste using a one-way ANOVA. *Parabasalia* abundances were 10-fold fewer in the winged alate class, compared to the worker caste [*F*_(3,36)_ = 11.9, *p* < 0.0001]. Protists belonging to the order *Oxymonadida* were less abundant in the winged alates and de-winged alates [*F*_(3,36)_ = 36.94, *p* < 0.0001]. We were interested in determining if the abundance of bacterial OTUs correlated with the abundance of protists and tested for this by calculating Pearson correlations between each protist group and the *Treponema* (*Spirochaetes*) or *Endomicrobia* OTUs. The two-tailed *p*-values were Bonferroni-corrected to account for the number of comparisons performed. Twenty-three of the 49 *Treponema* OTUs correlated with the *Oxymonadida* protists, and only ten of forty-nine correlated with the *Parabasalia* (*p* < 0.001, **Supplementary Figure 4A**). Of these nine *Treponema* OTUs were identical matches for sequences found in the DictDb database and belong to *Treponema* subgroups Ia, Ic, and Ig the OTU IDs are listed (**Supplementary Figure 4A**). Eight out of twelve *Endomicrobia* correlated with *Parabasalia*, and seven of 12 correlated with *Oxymonadida* protists (*p* < 0.004, **Supplementary Figure 4B**).

## Discussion

### The *R. flavipes* Core Microbiota

The presence of a core microbiota and its composition provides insight into the structure of the microbial community in the habitat of interest, and suggests the metabolic potential and conserved functions of the community (Tap et al., 2009; Huse et al., 2012; Shade and Handelsman, 2012). In termites, many groups have researched the hindgut microbial population, and the presence of a core community has been suggested (Fisher et al., 2007; Boucias et al., 2013; Huang et al., 2013; Scharf, 2015), however, the actual core microbiota remains to be defined as previous groups relied on pools of animals in their analysis which hides the variation amongst individuals. The common microbiota present in nine species of fungus-growing termites was determined, which included 42 taxa comprising eight phyla. The majority of sequences were assigned to two taxa, *Bacteroidetes* and *Firmicutes* (78.6% of sequences) (Otani et al., 2014). In a large survey, Dietrich et al. pooled gut homogenates from 3 to 10 individuals and reported the similarities and differences of the gut microbiota among cockroaches, lower termites, and higher termites. That study found that between 77 and 79% of the sequenced reads, and between 50 and 87 genus-level taxa were assigned to the shared microbiota in each of the three groups tested, cockroaches, lower termites, and higher termites (Dietrich et al., 2014).

For determining the core microbiota, we optimized the DNA extraction protocol for working with individual hindguts. This optimization allowed us to determine variation between individuals when calculating the core microbiota. Determining the composition of the hindgut microbiota from 45 termites obtained from seven different colonies (up to 250 km apart) aided in determining a taxonomic and OTU-based core community (**Table 1**). The more abundant taxa that we identified to comprise the core are identical to the phyla previously reported in the hindgut of *R. flavipes: Spirochaetes*, *Elusimicrobia*, *Bacteroidetes*, *Firmicutes*, and *Proteobacteria* (Fisher et al., 2007; Ohkuma, 2008; Boucias et al., 2013; Huang et al., 2013). Included in these phyla are the abundant taxa, *Treponema*, *Endomicrobia*, and *Azobacteroides*. Less abundant taxa comprising the core include *Desulfovibrio*, *Lactococcus*, *Bacteroidales*, which have been reported to be present in the hindgut of termites (Scharf, 2015). While future studies that expand the geographic range could reduce the number of OTUs comprising the core, the large number of animals and geographic range sampled provides an excellent baseline.

The bacterial, protist, and archaeal populations in the wood-feeding termite hindgut are known to play an integral part in the digestion of the wood meal, and without the complex bacterial community, the termite cannot survive (Raina et al., 2004; Rosengaus et al., 2011). The presence of a core microbiota suggests that each member of the core fills a niche in the termite hindgut that is consistently present despite changes in habitat, geography or food source and thus are likely to contribute to the overall health of the termite. We hypothesize that in *R. flavipes*, the core microbiota is made up of 69 OTUs in 16 taxa and accounts for more than 67.05% of the sequences. The dominant taxa found in the core were *Treponema* (41.43%) and *Endomicrobia* (18.50%), and were reported as part of the common microbiota by Dietrich et al (Dietrich et al., 2014). *Treponema* is a protist ectosymbiont as well as a free-living bacterium in the lumen of the hindgut and is the primary producer of acetate via reductive acetogenesis, which is the main nutrient for the termite host (Leadbetter et al., 1999; Graber and Breznak, 2004). *Endomicrobia* have been found to exist as a protist endosymbiont and a free-living bacterium in the hindgut, providing vitamins, and amino acids (Hongoh et al., 2008; Ikeda-Ohtsubo et al., 2010). The genus *Azobacteroides* (3.44%) was also found in the core and has been previously identified as a protist symbiont and nitrogen fixer in the gut of the termite *Coptotermes formosanus* (Raina et al., 2004). Each of the less abundant taxa were comprised of 1-2 OTUs and each accounted for less than 1% of the total sequences but were present in over 95% of the samples. In general, the abundances of the different taxa comprising the core followed similar patterns in the different colonies analyzed in this study (**Figure 1**). The core taxa were determined using colonies CT.B, CT.C, CT.D, MA.A, MA.B, and MA.C because they were represented by single hindgut samples. Although colony CT.A was not used in the calculation of the core microbiota due to pooled sampling methods, the taxa found in the core were found in similar abundances (**Figure 1**). The consistent detection of 13 taxa that each accounted for less than 1% of the sequences suggests an important, yet still undefined role for these low abundant organisms.

Sequencing depth will also affect the core size, as a greater sequencing depth increases the chance detection of less abundant taxa. By analyzing 15,000 sequences per sample, we had a greater likelihood of including these taxa in the core as opposed to utilizing fewer sequences, which may only detect low abundant taxa sporadically. This has important implications for previous studies of other microbial communities. The greater sampling depth provided by Illumina sequencing is likely to show a larger core microbial community by detecting less abundant sequences in different environments. Sixteen OTUs that we identified in this study were also present in the DictDb cockroach and termite symbiont reference database, which does not include data from *R. flavipes* but includes data from two related species, *R. chinensis* and *R. speratus* (Mikaelyan et al., 2015). A *Treponema* OTU was present at 3% in one sample, and another at 2% in one sample. The rest of the 14 OTUs were present at 1% or less in all samples. It is interesting that the most abundant OTUs were species-specific at least in this case. It has been reported that the hindgut protists vary depending on termite species (Ohkuma, 2008) and this may be the case for hindgut bacteria as well and has been suggested by Dietrich et al. (2014).

### Analysis of the Hindgut Microbiota in Termites from Different Colonies

The microbiota has been shown to aid in host health when present in a symbiotic relationship. The maintenance of the bacterial community throughout a colony of bumble bees aids in the protection from the parasite *Crithidia bombi* (Koch and Schmid-Hempel, 2011). Rosengaus et al. (2011) reported the importance of the hindgut microbiota on host survival in the dampwood termite, *Zootermopsis angusticollis* and *R. flavipes.* In that study, a 64% reduction in bacterial diversity and a small, short-term reduction of gut protists occurred when the diet was supplemented with 0.005 g/mL of the antibiotic, Rifampin. Lower survival rates and a reduction of eggs, larvae, and soldiers were observed for both termite species and correlated with the reduced bacterial diversity (Rosengaus et al., 2011). In addition to proctodeal feeding, it has been shown that social grooming and deposition of fecal contents and saliva in foraging galleries spread termite hindgut bacteria throughout a colony. The bacteria (mostly *Actinobacteria* sp.) in these galleries have been shown to breakdown the cell walls of pathogenic fungi (Klassen, 2014) and possibly pathogenic bacteria (Carr et al., 2012). The maintenance of the *R. flavipes* hindgut microbiota within a colony could provide the termite with protection from microbial invaders in addition to the provision of nutrients.

Previous studies have characterized the bacterial taxonomic abundances in *R. flavipes* using the V1–V3 and V5–V6 region (Boucias et al., 2013; Huang et al., 2013; Nelson et al., 2014), and our study with samples from seven colonies in Connecticut and Massachusetts revealed similar taxonomic composition and abundances. The most abundant phyla represented were *Spirochaetes* (∼55%), *Elusimicrobia* (∼24%), and *Bacteroidetes* (∼10%), with lower abundant representatives from the phyla *Firmicutes*, *Proteobacteria*, *Tenericutes*, *Synergistes*, and unclassified *Bacteria*. The relative abundance of sequences in the termite hindgut varied slightly depending on which region of the 16S rRNA gene was sequenced, which is a known caveat of 16S rRNA studies (Janda and Abbott, 2007; Aird et al., 2011; Soergel et al., 2012; Nelson et al., 2014) but did not change the overall composition.

Different colonies in the same geographic area have been suggested to harbor slightly different hindgut microbiotas, which might allow termites to distinguish nest mates from invaders (Hongoh et al., 2005; Minkley et al., 2006). Beta diversity analyses in this study are consistent with this concept of nest specificity as termites within a colony show a greater similarity of the hindgut microbiota than to other colonies. Colonies CT.A and CT.C were sampled over 4 months and each grouped as a colony according to the NMDS plot. This shows the homogenous nature and stability of the hindgut microbiota within a colony that was transferred from the field and maintained in the laboratory. It was interesting to note, however, that these colonies had a lower Shannon Index (H’) which is indicative of a lower OTU richness compared to the other colonies. The Shannon Equitability (E_H_) was also lower in these two colonies which indicates more evenness compared to the other colonies. The lower richness and lower evenness may be due to the colonies being kept in the lab, whereas the other colonies were sampled directly from their natural habitat, which would be analogous to a “zoo” effect (Ley et al., 2008; Kohl and Dearing, 2014). A Bray–Curtis analysis of the samples in this study showed hindgut microbiotas from the same colony grouping together (**Figure 3**). Colony MA.C from Woods Hole, MA, shows the most differences according to relative abundances of multiple taxa among the colonies (**Figure 2**), however, our analysis of the COII sequences did not reveal a corresponding phylogenetic difference of the hosts. This may be due to environmental conditions such as higher salt concentration in the air, sand-rich soils, and the lack of dense forestry. Leadbetter and Breznak (1996) reported different morphotypes of methanogens found in *R. flavipes* hindguts in Michigan and Woods Hole, MA, USA, which coincides with our findings of differing bacterial taxa. While the core taxonomic abundances show very small differences, it can be that changes in the relative abundance of key taxa or the fluctuation of low abundance taxa between samples, which could be involved in colony recognition.

### The Hindgut Microbiota Among Different Castes

While workers are the primary caste many researchers study, the soldier and alate castes play important roles in the colony. Soldiers protect the colony from invaders and cannot morph into any other caste. Alates, a form of reproductive termite, swarm to a new area to establish a new colony, wherein they will become the primary reproductives (king and queen). During the transition to winged alates, the termites lose the majority of their gut protists and rely on lipids and glycogen stored in the fat body for nutrition (Costa-Leonardo et al., 2013). Alates shed their wings after swarming to an area to start a new colony, morph into primary reproductives and give rise to juvenile termites. The primary reproductives forage on wood, feed the first generation of juveniles until they are ready to provide for the colony and transmit the symbionts to the juveniles unless some symbionts are acquired from the environment (Shimada et al., 2013). Lewis et al demonstrated that protist abundances in the hindgut differ depending on the feeding habits of the caste, with protist abundances being lower in the alate and soldier castes of three *Reticulitermes* species (Lewis and Forschler, 2013). This finding leads to the question of whether or not different castes with different digestive functions harbor the same hindgut bacterial community.

The dramatic drop in abundance of the protozoal symbionts *Spirochaetales* and *Endomicrobia* in the winged alates coincides with the dramatic decrease in protist numbers during this morphing stage, as shown by Shimada et al. (2013). During this time, the termite is building up fat bodies and storing more nutrients in the fat bodies as the animals are preparing to swarm and establish a new colony (Shimada et al., 2013). We performed qPCR to quantify the two protist groups found in the hindgut, *Parabasalia* and *Oxymonadida* on the same hindgut samples that we sequenced the 16S rRNA gene from. These data show a drop in abundance of both protist groups in the winged (*Parabasalia* and *Oxymonadida*) and dewinged alates (*Oxymonadida*), when compared to the worker caste. When evaluating whether OTUs were potential protist symbionts, seven and eight of the 12 *Endomicrobia* OTUs correlated with *Oxymonadida* and *Parabasalia* protists, respectively (**Supplementary Figure 4B**). *Endomicrobia* exists in the hindgut as a strict endosymbiont in both *Oxymonadida* and *Parabasalia* protists (Ohkuma et al., 2007), which likely accounts for the large percentage of OTUs correlating with either protist group. The *Endomicrobia* OTUs that do not correlate with the abundance of either protist group could be due to sequencing errors, PCR sensitivity, being present inside protists that do not change abundance according to caste differentiation or exist without an obligate association with protists. The *Treponema* OTUs correlate with the *Parabasalia* and *Oxymonadida* as well (10/49 and 23/49, respectively). *Treponema* are known to exist in the hindgut as protist symbionts as well as free-living, which could account for less than half of the OTUs actually correlating with either protist group (**Supplementary Figure 4A**) (Leadbetter et al., 1999). Using the DictDb database, OTUs belonging to the *Treponema* taxon were further classified into subgroups (*Treponema Ia, Ib, Ic, Ig, II*) and this revealed that *Treponema Ia* was the most abundant taxa (**Figure 5**). *Treponema Ib* was the least abundant among the five groups and was also consistent among the castes, suggesting that this group may be a free-living spirochete. The abundance of *Treponema Ia* and *Treponema II* was significantly lower in winged alates compared to the worker caste, suggesting that these may be protist symbionts. Sequences corresponding to the order *Bacteroidales* nearly doubled in abundance in both the winged and de-winged alates as compared to workers and soldier microbiotas, which could be a result from a greater growth rate or of the *Spirochaetales* and *Endomicrobia* sequences dropping in abundance as these are not absolute but relative values. The spike of *Enterobacteriales*, *Flavobacteriales*, and *Pseudomonadales* sequences in the winged alates suggests the hindgut is in an altered state in the winged alates, which may reflect the physiological needs of the alate during swarming.

### Overall Characterization of the *R. flavipes* Hindgut Microbiota

Studying the hindgut microbiota of individual termites from multiple colonies and castes has added to the understanding of the bacterial components of this complex symbiosis. The ability to sequence many hindgut samples has allowed for a more comprehensive comparison of different colonies and various castes, as well as the determination of a core microbiota in the *R. flavipes* species. Defining a core microbiota in the *R. flavipes* hindgut has revealed the presence of relatively constant and complex bacterial populations in the hindgut of workers. The differences in the composition of the bacterial and protist communities in the winged alates and de-winged alates suggest that major changes occur in the termite digestive tract physiology in this caste, perhaps related to the animals not feeding while relying on the fat body, which would lead to “starvation” of the protists, bacteria and archaea in the hind gut. Importantly, the community cannot be depleted of the core members, as it needs to be passed on to the workers from the new colony unless they are acquired from the environment. The maintenance of such a large core community is important as it suggests that a consistent group of microorganisms participates in the complex degradation of lignocellulose in the hindgut and the provision of nutrients that this simple diet is depleted in. The termite holobiont, or the combination of host and symbionts, which together form a functional unit, is complex and likely to be even more complex as insight is gained into viruses or fungi that may be present inside the termite in addition to the archaea, bacteria, and protists (Rohwer et al., 2002; Zilber-Rosenberg and Rosenberg, 2008; Bordenstein and Theis, 2015).

Studying the bacterial and protist populations in the *R. flavipes* hindgut throughout different life stages, colonies, and over time provides a cohesive representation of the community dynamics. The consistent presence of sequences at low percentages suggests that these analyses need to be done with sufficient sensitivity to detect the activities of these members as well, albeit technical caveats make the analysis of less abundant or even rare taxa more challenging (Reeder and Knight, 2009). As the bacteria in the hindgut are not easily cultured outside the host, the ability to manipulate the hindgut community as a whole *in vivo*, for example through environmental changes or dietary changes, allows for this host to become a model for complex symbioses by revealing principles that are conserved among distantly related digestive tract symbioses (Ruby, 2008; Nelson and Graf, 2012; Maltz et al., 2014).

## Author Contributions

JB performed the laboratory work and statistical analysis. JB and JG contributed the experimental design and writing of the manuscript.

## Conflict of Interest Statement

The authors declare that the research was conducted in the absence of any commercial or financial relationships that could be construed as a potential conflict of interest.

## Footnotes

1 https://github.com/jstjohn/SeqPrep

2 www.graphpad.com
