# Supplementary material for: Characterization of the Core and Caste-Specific Microbiota in the Termite, Reticulitermes flavipes
Source: Front Microbiol. 2016 Feb 17;7:171. doi: 10.3389/fmicb.2016.00171 (PMC4756164; doi:10.3389/fmicb.2016.00171)
Supplement: Supplementary file 2 [file Supplemental_Figures.PDF]

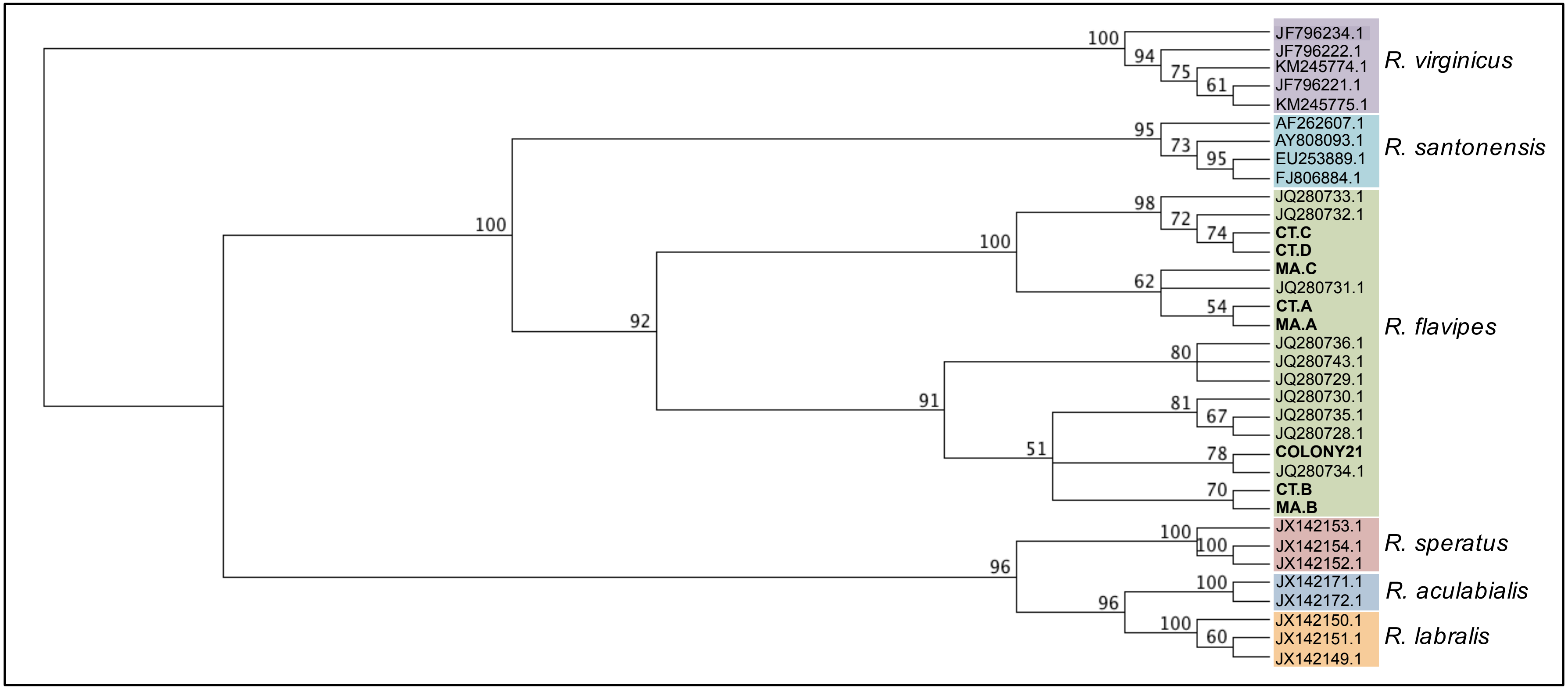

Supplementary Figure 1. Neighbor-joining (NJ) Analysis of Cytochrome Oxidase II (COII) Gene Sequences. COII DNA sequence data from termites used in this study and *Reticulitermes* species obtained from NCBI were used in NJ analysis with 100 bootstrap iterations. Sequences from R. virginicus were used to root the phylogram. Numbers above the nodes indicate NJ bootstrap support.

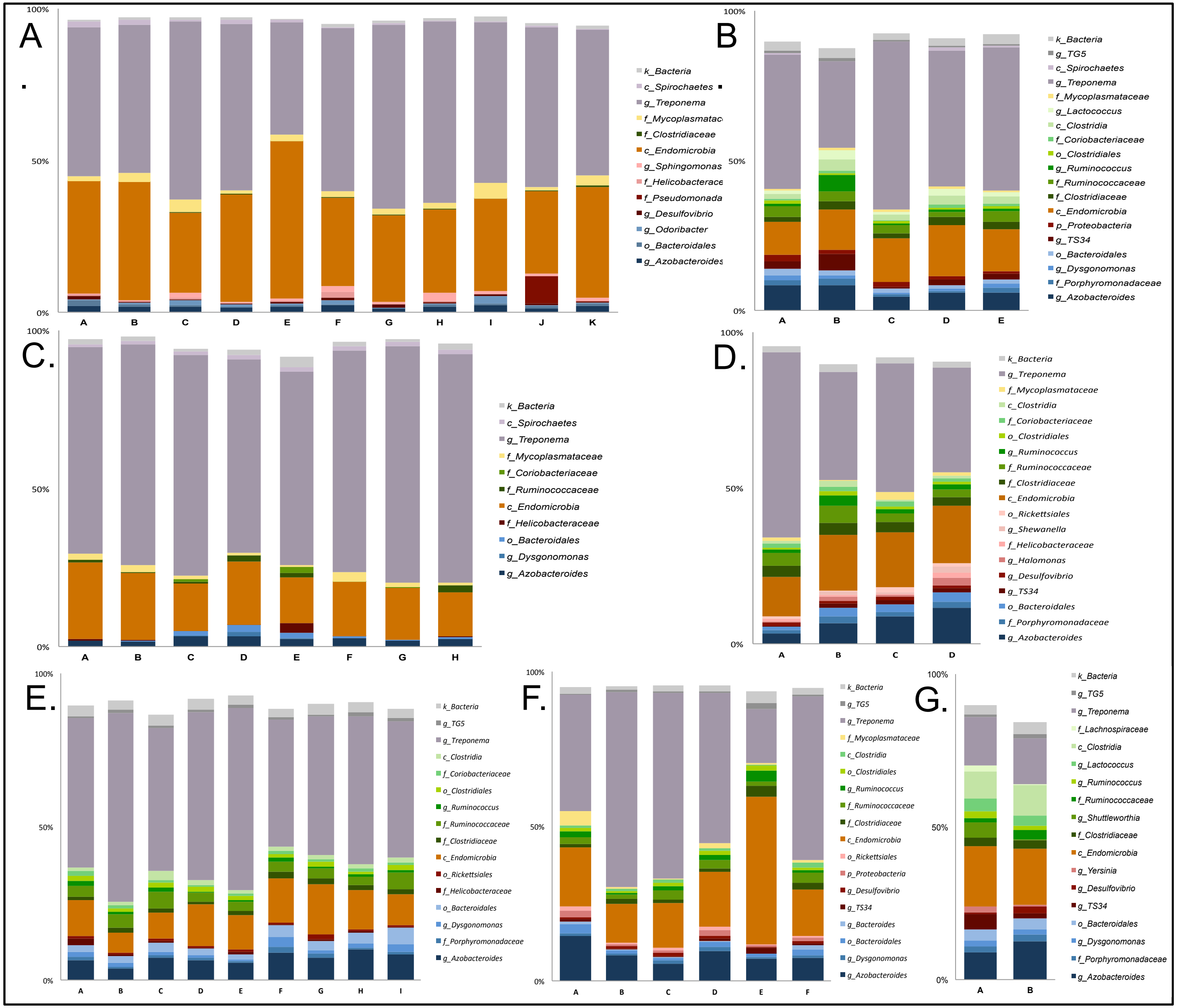

Supplementary Figure 2. Composition of the Hindgut Microbiota in Connecticut (CT) and Massachusetts (MA). Connecticut and Massachusetts colonies do not differ by state of collection, but vary slightly between colonies. Colonies, CT.A (A), CT.B (B), CT.C (C), and CT.D (D). Colonies CT.A and CT.C are lacking most of the *Firmicutes* phylum and a larger abundance of the *Spirochaetes*. These colonies were lab-maintained for over a year, while the other colonies were sampled immediately upon collection. MA.A (F), MA.B (F), and MA.C (G). Colony MA.C has a lower abundance of *Spirochaetes* than the other Massachusetts colonies and a greater taxonomic diversity.

Abundance																			
Wokrer A	Worker B	Worker C	Worker D	Worker E	Worker F	Worker G	Worker H	Worker I	Soldier A	Soldier B	Soldier C	Soldier D	Soldier E	Winged Alate A	Winged Alate B	Dewinged Alate A	Dewinged Alate B	Dewinged Alate C	
31.19%	72.57%	52.09%	60.37%	48.11%	48.82%	54.07%	51.20%	17.39%	63.03%	80.74%	67.24%	63.56%	75.93%	22.76%	0.53%	28.33%	32.13%	32.66%	*o_Spirochaetales*
5.06%	14.13%	16.18%	28.50%	17.27%	26.38%	29.53%	13.87%	47.12%	25.95%	9.44%	19.05%	17.95%	9.47%	2.19%	0.05%	14.68%	17.25%	20.23%	*c_Endomicrobia*
26.20%	3.11%	12.02%	1.77%	13.54%	8.62%	4.56%	15.13%	9.74%	4.60%	1.70%	5.45%	4.46%	3.56%	44.68%	22.77%	25.31%	17.74%	18.94%	*o_Bacteroidales*
8.89%	3.33%	7.93%	1.23%	7.41%	2.83%	1.20%	4.22%	10.91%	0.35%	1.09%	0.86%	3.33%	3.53%	3.33%	0.23%	3.21%	1.88%	4.03%	*o_Clostridiales*
3.01%	1.90%	2.04%	0.72%	1.99%	2.23%	1.16%	1.63%	3.86%	0.39%	2.39%	0.55%	0.74%	0.75%	2.14%	0.04%	2.46%	1.73%	1.53%	*k_Bacteria*
5.07%	0.42%	1.56%	1.12%	1.69%	1.29%	0.74%	2.94%	0.64%	1.65%	0.40%	1.66%	0.58%	1.52%	5.81%	0.08%	1.52%	0.99%	2.21%	*o_Desulfovibrionales*
2.72%	0.49%	0.85%	0.51%	1.98%	0.84%	0.66%	1.57%	0.47%	0.38%	0.51%	0.49%	0.67%	0.35%	2.40%	0.01%	0.58%	0.99%	0.08%	*p_Proteobacteria*
0.40%	0.63%	0.78%	1.97%	1.90%	0.17%	1.67%	0.51%	0.47%	0.54%	1.66%	1.04%	1.07%	0.19%	0.05%	0.01%	0.04%	0.77%	0.21%	*o_Mycoplasmatales*
2.24%	0.18%	0.72%	0.13%	0.46%	0.45%	0.10%	2.20%	1.80%	0.21%	0.08%	0.21%	0.11%	0.37%	1.90%	0.17%	0.36%	0.29%	0.25%	*o_Synergistales*
2.05%	0.46%	1.32%	0.18%	0.92%	1.56%	0.09%	1.15%	0.42%	0.02%	0.08%	0.17%	0.41%	0.25%	2.05%	0.01%	3.89%	0.60%	0.54%	*c_Clostridia*
2.74%	0.23%	0.05%	0.06%	0.18%	0.89%	1.86%	1.99%	0.10%	0.07%	0.28%	0.39%	0.90%	0.30%	0.18%	0.03%	0.50%	1.08%	3.28%	*o_Campylobacterales*
1.50%	0.06%	0.75%	0.03%	0.78%	1.54%	0.26%	0.51%	1.96%	0.03%	0.04%	0.25%	0.16%	0.29%	0.82%	0.73%	8.14%	7.20%	6.04%	*o_Rhodocyclales*
0.19%	0.21%	0.72%	0.80%	1.27%	1.11%	0.96%	0.26%	0.41%	0.93%	0.22%	0.97%	0.64%	0.34%	0.03%	0.00%	0.11%	0.23%	0.35%	*o_Rickettsiales*
2.79%	0.06%	0.49%	0.23%	0.44%	0.37%	0.02%	0.67%	0.19%	0.00%	0.01%	0.09%	0.61%	0.33%	0.40%	0.01%	0.31%	0.37%	0.18%	*o_Coriobacteriales*
0.06%	0.39%	0.62%	0.36%	0.45%	0.13%	0.11%	0.08%	1.37%	0.04%	0.17%	0.00%	0.68%	0.40%	0.01%	0.01%	0.04%	0.14%	0.26%	*c_Actinobacteria*
0.64%	0.08%	0.52%	0.07%	0.43%	0.50%	0.11%	0.14%	1.01%	0.12%	0.05%	0.42%	0.27%	0.15%	1.54%	6.99%	1.37%	0.41%	0.82%	*o_Lactobacillales*
0.00%	1.24%	0.00%	0.65%	0.02%	0.17%	0.07%	0.00%	0.01%	0.07%	0.74%	0.83%	1.10%	0.97%	0.00%	0.00%	0.11%	0.43%	0.03%	*c_Spirochaetes*
0.30%	0.06%	0.20%	0.22%	0.33%	0.12%	0.38%	0.43%	0.13%	0.51%	0.07%	0.12%	0.28%	0.13%	0.06%	0.01%	0.06%	0.11%	0.13%	*o_HA64*
1.24%	0.09%	0.06%	0.00%	0.01%	0.02%	0.05%	0.14%	0.02%	0.00%	0.06%	0.05%	0.09%	0.02%	5.78%	0.00%	0.01%	0.06%	0.05%	*p_Bacteroidetes*
0.23%	0.07%	0.01%	0.03%	0.03%	0.09%	0.11%	0.16%	0.01%	0.18%	0.09%	0.04%	0.03%	0.08%	0.12%	0.00%	0.07%	0.43%	0.06%	*c_Rs-J96*
0.10%	0.01%	0.03%	0.06%	0.02%	0.01%	0.25%	0.08%	0.04%	0.05%	0.02%	0.03%	1.08%	0.33%	0.04%	0.04%	0.08%	0.04%	0.04%	*o_Oceanospirillales*
0.20%	0.00%	0.06%	0.00%	0.10%	0.01%	0.00%	0.16%	0.00%	0.02%	0.01%	0.00%	0.00%	0.29%	1.01%	18.40%	0.00%	0.00%	0.00%	*o_Enterobacteriales*
0.19%	0.00%	0.10%	0.03%	0.04%	0.03%	0.01%	0.12%	0.01%	0.00%	0.00%	0.00%	0.00%	0.00%	0.92%	0.00%	0.13%	0.40%	0.04%	*o_RF32*
0.01%	0.00%	0.02%	0.03%	0.02%	0.02%	0.15%	0.02%	0.01%	0.05%	0.01%	0.01%	0.29%	0.06%	0.03%	2.96%	0.04%	0.03%	0.05%	*o_Burkholderiales*
0.10%	0.00%	0.00%	0.00%	0.00%	0.02%	0.00%	0.04%	0.00%	0.00%	0.00%	0.00%	0.00%	0.00%	0.89%	1.08%	0.40%	0.20%	0.09%	*o_RsaHF231*
0.01%	0.00%	0.00%	0.00%	0.00%	0.00%	0.00%	0.11%	0.00%	0.00%	0.00%	0.00%	0.00%	0.01%	0.05%	27.16%	0.00%	0.00%	0.00%	*o_Flavobacteriales*
0.02%	0.00%	0.00%	0.01%	0.00%	0.00%	0.03%	0.04%	0.00%	0.11%	0.00%	0.00%	0.00%	0.01%	0.04%	18.10%	0.01%	0.02%	0.01%	*o_Pseudomonadales*
0.00%	0.00%	0.02%	0.00%	0.01%	0.03%	0.00%	0.01%	0.00%	0.01%	0.00%	0.01%	0.02%	0.02%	0.06%	0.61%	8.08%	14.41%	7.52%	*o_Neisseriales*

0% 80%

Supplementary Figure 3. Taxonomic abundances of the hindgut microbiota in the worker, soldier, winged alate, and de-winged alate castes. Each termite used in the caste analysis (Figure 3) is shown.

A.

OTU ID GG/DictDb[a]	Taxonomic Subgroup[b]	*Parabasalia* P- value[c]	*Parabasalia* R squared	*Oxymonadida* P- value[c]	*Oxymonadida* R squared
		Treponema			
77905/UltTr169	*Treponema Ia*	0.0003	0.312	< 0.0001	0.4525
77925/UltTr173	*Treponema Ia*	0.0011	0.259	< 0.0001	0.4656
78075/UltSpi49	*Treponema Ia*	0.0277	0.1276	< 0.0001	0.3946
114589/UltB4827	*Treponema Ia*	0.0001	0.3367	< 0.0001	0.4822
114698/UltB4828	*Treponema Ia*	0.0063	0.1897	< 0.0001	0.4807
135860/UltTr348	*Treponema Ic*	< 0.0001	0.4258	< 0.0001	0.6102
4116/UltTr183-4	*Treponema Ig*	< 0.0001	0.4105	< 0.0001	0.6876
114560/UltB4833	*Treponema Ig*	0.0007	0.2786	< 0.0001	0.4183
143443/UltTr177	*Treponema Ig*	0.002	0.2362	< 0.0001	0.4175
77582		0.0006	0.2799	< 0.0001	0.4162
114660		0.0026	0.2246	< 0.0001	0.4323
141454		0.0046	0.2028	< 0.0001	0.4145
denovo136320		0.0002	0.3152	< 0.0001	0.3913
denovo15637		0.0089	0.1754	< 0.0001	0.4054
denovo193592		0.0218	0.1377	0.0001	0.336
denovo208664		0.0004	0.2939	< 0.0001	0.4391
denovo255921		0.0001	0.3387	< 0.0001	0.4327
denovo38396		0.0145	0.1548	0.0008	0.2727
denovo44029		0.0297	0.1247	0.0004	0.302
denovo53179		0.0003	0.3052	< 0.0001	0.5813
denovo67757		0.0694	0.08867	0.0004	0.2939
denovo84958		0.0071	0.1848	< 0.0001	0.3533
denovo94306		0.0124	0.1613	0.0001	0.3374

B.

OTU ID	*Parabasalia* P-value[d]	*Parabasalia* R squared	*Oxymonadida* P-value[d]	*Oxymonadida* R squared
		Endomicrobia		
114693	< 0.0001	0.427	< 0.0001	0.5298
114701	0.0024	0.2287	0.0005	0.2879
114552	< 0.0001	0.3531	0.0047	0.2011
108758	0.0162	0.1503	0.0085	0.1772
114047	0.0344	0.1183	0.0091	0.1742
15539	0.0203	0.1408	0.0065	0.1884
194402	0.0002	0.3315	< 0.0001	0.4248
30026	0.0007	0.2779	< 0.0001	0.365
36316	0.0016	0.2453	0.0002	0.3197
42156	0.0001	0.3405	0.0001	0.3402
87158	0.3475	0.02455	0.1896	0.0473
89039	0.0005	0.289	0.001	0.2613

[a]OTU ID from the Greengenes and DictDb databases
[b]Taxonomic classification based on the DictDb database
[c]A p-value <0.001 is significant of a correlation.
[d]A p-value <0.004 is significant of a correlation.
- Gray-shaded OTUs are not significantly correlated.

Supplementary Figure 4. Correlation of *Treponema* and *Endomicrobia* Bacterial OTUs to *Parabasalia* and *Oxymonadida* Protists. *Treponema* and *Endomicrobia* correlate with *Parabasalia* and *Oxymonadida* protists. *Treponema* and *Endomicrobia* OTUs from the core microbiota were compared to the protist qPCR data using a Pearson correlation. R squared values were calculated and the p-values were Bonferroni corrected. Areas shaded in gray represent OTUs that did not significantly correlate with a protist. The *Treponema* and *Endomicrobia* OTUs are considered significantly correlated if p<0.001 and p<0.004 respectively.
